# Lung density on high resolution computer tomography (HRCT) reflects degree of inflammation in smokers

**DOI:** 10.1186/1465-9921-15-23

**Published:** 2014-02-24

**Authors:** Reza Karimi, Göran Tornling, Helena Forsslund, Mikael Mikko, Åsa M Wheelock, Sven Nyrén, Carl Magnus Sköld

**Affiliations:** 1 Department of Medicine and Center for Molecular Medicine (CMM), Karolinska Institutet, Lung-Allergy Clinic N2:02, Karolinska University Hospital Solna, Stockholm, SE 171 76, Sweden; 2 Department of Molecular Medicine and Surgery, Karolinska Institutet, Karolinska University Hospital Solna, Stockholm, Sweden

**Keywords:** Inflammation, Attenuation, Lung density, Smoking, CT, Lung function, Gender, Bronchoalveolar lavage

## Abstract

**Background:**

Smokers have increased cell concentration in the lower respiratory tract indicating a chronic inflammatory state, which in some individuals may lead to development of chronic obstructive pulmonary disease (COPD). Computer tomography (CT) imaging provides means of quantifying pulmonary structure and early signs of disease. We investigated whether lung density on high resolution CT differs between smokers and never-smokers and if this were associated to intensity of inflammation.

**Methods:**

Forty smoking volunteers with normal pulmonary function, 40 healthy never-smokers and 40 patients with COPD of GOLD stage I-II, were included. Mean lung attenuation and percentage of pixels in the lung with attenuation between −750 and −900 HU (percentage higher density spectrum (%HDS)) were calculated on inspiratory CT-scans. Markers of systemic inflammation in blood and cell counts in bronchoalveolar lavage (BAL) fluid were recorded.

**Results:**

Lung density expressed as %HDS was increased in smokers (44.0 ± 5.8%) compared to both never-smokers (38.3 ± 5.8%) and patients with COPD (39.1 ± 5.8%), (p < 0.001, for both). Females had denser lungs than males, which was dependent on body height. Cell concentration in BAL were correlated to lung density in smokers (r = 0.50, p < 0.001).

**Conclusions:**

Lung density on CT is associated with cell concentration in BAL in smokers and may mirror an inflammatory response in the lung. Gender difference in lung density is dependent on height. In COPD with emphysema, loss of lung tissue may counterbalance the expected increase in density due to inflammation. The findings may help to interpret high resolution CT in the context of smoking and gender and highlight the heterogeneity of structural changes in COPD.

## Introduction

Cigarette smoke induces an inflammatory reaction in the lung and is a major risk factor for a number of lung diseases such as chronic obstructive pulmonary disease (COPD) and diffuse parenchymal lung diseases [1]. Further, cigarette smoking leads to elevation of both cells and soluble markers of systemic inflammation in the circulation [2]. Infiltration of macrophages and mononuclear cells in the lung leads to tissue damage and release of numerous inflammatory mediators resulting in an increased epithelial permeability and oedema in the lung interstitium [3]. Both local and systemic inflammation in smokers may be present before any significant clinical symptoms appear [2].

Computer tomography (CT) imaging of the lung can non-invasively detect and quantify lung abnormalities [4-7]. Early changes in airways and lung parenchyma may be recognized in smoking individuals with normal pulmonary function before any signs of lung function impairment [8,9]. In CT scans, attenuation is measured by Hounsfield Units (HU), where attenuation for water is defined as 0 HU and air as −1000 HU [10]. Several authors have focused on the region of the lung with low attenuation i.e. <−910 or −950 HU as a quantitative assessment of emphysema. These studies are usually performed on subjects with advanced emphysema or on heavy smokers included in lung cancer screening programs [10-12]. Despite the potential of CT to non-invasively detect and quantify early subclinical pathological changes such as increased density, studies in this field are scarce.

Given the intense systemic and local inflammation induced by smoking, we asked whether this may be mirrored by alterations in the high density spectrum assessed by high resolution CT. We therefore hypothesized that lung density were associated with an inflammatory response, and to test this a group of smokers with normal pulmonary function, a matched group of healthy never-smokers and a group of patients with COPD (GOLD I-II) underwent inspiratory CT examinations. The percentage of the lung parenchyma in the high density spectrum, i.e. with attenuation between −750 to −900 HU (%HDS) was calculated within pre-defined thresholds. We also analysed blood samples and performed bronchoscopy and bronchoalveolar lavage for cell concentrations and differential cell counts as measures of systemic and local inflammation.

## Materials and methods

### Subjects

The study was performed as a part of the Karolinska COSMIC study [13,14] comprising 120 individuals in the age 45–65 years and matched for gender (20/20 per group) consisting of healthy never-smokers, smokers with normal lung function, and COPD patients (GOLD, I-II). Of the COPD patients, 28 were current smokers and 12 ex-smokers with a period since smoking cessation of more than 2 years. A medical examination and a posteroanterior and lateral chest X-ray were performed. Subjects with any significant medical condition or lung parenchymal abnormality except COPD on the chest X-ray or CT- scans were excluded. Subjects with asthma, allergy or airway infection were not included, and no one used inhaled or oral corticosteroids or had an exacerbation during at least 3 months prior to inclusion. All subjects completed a self-administered questionnaire (Chronic Respiratory Questionnaire, CRQ). Blood samples were obtained by venipuncture, and high sensitive C-reactive protein (CRP), orosomucoid, haptoglobin, immunoglobulin G and white blood cells counts were analysed according to routine standard methods at the Department of Clinical Chemistry, Karolinska University Hospital, Stockholm, Sweden.

### Lung function

Forced expiratory volume in one second (FEV_1_) and forced vital capacity (FVC) were measured 20 minutes after bronchodilatation with two inhalations of 0.5 mg Terbutalin (Bricanyl® Turbuhaler®; AstraZeneca, Södertälje, Sweden) using a spirometer (Vmax 229–6200 Legacy, USA). Total lung capacity (TLC) and residual volume (RV) were measured by body plethysmography. Carbon monoxide diffusing capacity (DLco) was measured by the single breath method and corrected for haemoglobin. Measurements were expressed as percentage of predicted values according to the European Community of Coal and Steel [15,16].

### Computed tomography (CT) imaging

Non-contrast enhanced, multi-detector helical CT examinations were performed using a Siemens Somatom sensation 16 slice CT (Siemens, Erlangen, Germany). Scans were done in a supine position and covered the entire chest during full inspiration. The scanning parameters were 16 × 0.75 mm collimation, 120 kV, rotation time 0.5 s and pitch 1.5. Dose modulation was used resulting in a mean of 180 mA. Axial images with 1 mm thickness and 0.5 mm overlap were reconstructed with filtered back projection and a medium high spatial resolution algorithm (B60f) in a 512 × 512 matrix.

### Image analysis

CT images were analysed using a semiautomatic program (Volume, Siemens), which automatically recognizes the lung borders and segments it from surrounding thoracic tissues by selecting pixels within determined attenuation limits from a seeding point. Attenuation values between −300 to −1024 HU were considered to delimit the lung parenchyma from surrounding structures and larger arteries.

Mean lung attenuation and lung volume were measured automatically. The measured values are the mean of density of air in alveoli and the surrounding epithelial, capillary and extracellular matrix and small airways. Increased oedema and accumulation of the inflammatory cells would increase the density, and destruction of alveolar walls and loss of tissues such as in emphysema would decrease the density.

The lung tissue was then further divided by adding the pixels within pre-defined CT attenuation limits in volumes and was then expressed as percentage of the entire lung volume [4,17]. Volumes in the range -300 to -750 HU were considered to represent pixels including tissue such as small bronchi, vessels and borders with denser structures (partial volume effect) and were analysed separately. Since measurements of parenchymal inflammation were the objective of the study, we focused on the areas between -750 and -900 HU, designated % high density spectrum (%HDS), representing lung tissue in the denser normal range. The lower limit was set to −900 HU based on previous studies which have shown that attenuation values less than −900 HU corresponds to macroscopic emphysema both in pathological specimens and in visual assessment of CT-scans [18,19].

### Bronchoscopy and bronchoalveolar lavage

Bronchoscopy with bronchoalveolar lavage was performed in order to obtain cells from the distal airways. Bronchoscopy procedure and preparation of BAL cells were performed according to a standard procedure in our department described in detail previously [20]. Briefly, the bronchoscope was wedged in a middle lobe segment bronchus and 5 aliquots of phosphate-buffered saline of 50 mL each were instilled. The volume of the recovered BAL fluid was measured and the recovery percentage was calculated. A cell pellet was obtained after centrifugation, and cell counts were prepared using a Bürker Chamber (Marienfeld, Germany). Cytospin slides were analysed after staining with May-Grünwald Giemsa and 500 cells were counted. Results were reported as both total cell count and cell concentration.

### Statistical analysis

Data were tested for normality using Bartlett’s test and one way analysis of variance (ANOVA) was utilized for univariate statistical testing. Bonferroni adjustment was employed for multiple testing compare mean values between groups. Bivariate analysis was conducted with two-tailed unpaired *t*-test for continuous variables. The values were expressed as mean and SD. Univariate linear regression were employed to evaluate relationship between %HDS and mean lung attenuation with potential covariates. Only significant covariates were then included in a multiple linear regression model. Statistical significances was defined as p values <0.05. All statistical analysis were performed using Stata12 software (Stata Corp, College Station, TX, USA).

### Ethical statement

Signed informed consent was obtained from all volunteers after verbal and written information. The study was approved by the Regional Ethical Review Board in Stockholm Sweden (dnr. 2006/959-31/1) and the Radiation Protection Committee at the Karolinska University Hospital, Stockholm, Sweden.

## Results

### Subject characteristics

Demography, lung function, markers of systemic inflammation and BAL data are outlined in Table 1 and the COPD group divided into current smokers and ex-smokers in Table 2. The three investigated groups were well matched regarding body mass index (BMI) and height. The COPD patients were slightly older than the smokers, but the smoking history was similar. Criteria for chronic bronchitis [21] were fulfilled for eleven of the smokers and for nine of the COPD patients. Diffusion capacity for carbon monoxide (DLCO) was significantly lower in the COPD group and in the smoker group compared to the never-smokers. COPD patients had significantly higher CRQ dyspnoea score than smokers and never-smokers (p < 0.001 for both). The COPD ex-smokers had smoked less than the COPD current smoker group and had a higher body mass index (BMI). There were no significant differences between males and females regarding age, BMI, smoking history and pulmonary functions parameters (data not shown).

**Table 1:** Demographics, lung function and markers of systemic and local inflammation in never smokers, smokers and COPD patients

**Variables**	**Never smokers**	**Smokers**	**COPD**
N (male/female)	40 (20/20)	40 (20/20	40 (20/20)
Age	57.0 ± 7.0	54.0 ± 6.2	59.2 ± 5.3^¤^
Body mass index (kg/m^2^)	25.9 ± 3.7	24.3 ± 3.1	25.3 ± 4.1
Height (centimetres)	172 ± 10.0	172 ± 10.8	171 ± 7.6
Smoking history (pack years)	N/A	35.2 ± 12.4	38.0 ± 11
Cigarette/day (last 6 months)	N/A	17.8 ± 6.6	16.4 ± 6.4
FEV_1_ (% predicted)	118.4 ± 12.8	109.5 ± 11.9^$$^	78.9 ± 11.7^$$$, ¤¤¤^
FVC (% predicted)	101.2 ± 10.7	96.9 ± 11.1	88.3 ± 12^$$$, ¤¤¤^
FEV_1_/FVC	0.82 ± 5.4	0.78 ± 4.7	0.61 ± 6.4^$$$, ¤¤¤^
DLco (% predicted)	90.9 ± 10.8	77.9 ± 12.2^$$$^	66.7 ± 13.0^$$$, ¤¤¤^
TLC (% predicted)	106 ± 10.7	106.8 ± 11.1	108.2 ± 14.8
RV (% predicted)	102.1 ± 25.3	112.9 ± 22.5	138.0 ± 33.6^$$$, ¤¤¤^
Chronic bronchitis (% of individuals)	0	25^$$$^	23^$$$^
Dyspnea score (CRQ)	6.8 ± 0.5	6.4 ± 0.77	6.0 ± 0.85^$$$, ¤¤^
White blood cell count (10^9^/L)	5.7 ± 1.1	7.4 ± 1.6^$$$^	7.7 ± 1.9^$$$^
Serum-high sensitive-CRP (g/L)	1.2 ± 0.89	1.9 ± 1.8	3.0 ± 2.7^$$$, ¤¤^
Serum-orosomucoid (g/L)	0.69 ± 0.12	0.78 ± 0.18^$^	0.84 ± 0.16^$$$^
Serum-haptoglobin (g/L)	0.88 ± 0.37	1.2 ± 0.50^$$$^	1.4 ± 0.46^$$$^
Serum-immunoglobulin G (g/L)	11.1 ± 2.0	9.1 ± 1.8^$$$^	9.6 ± 1.8^$$$^
BAL cell concentration (10^6^/L)	121.0 ± 49.8	557.0 ± 230^$$$^	378.3 ± 277^$$$, ¤¤¤^
BAL macrophages (10^6^/L)	103.4 ± 40.3	535.4. ± 160^$$$^	358.0 ± 272^$$$, ¤¤¤^
BAL lymphocytes (10^6^/L)	15.6 ± 17.6	14.1 ± 12.0	13.2 ± 8.8
BAL neutrophils (10^6^/L)	1.8 ± 1.6	4.6 ± 5.3^$$^	4.1 ± 3.7^$$^
BAL eosinophils (10^6^/L)	0.27 ± 0.53	1.5 ± 3.0	2.5 ± 8.2
BAL recovery (%)	64.5 ± 11.8	58.4 ± 10.7	45.8 ± 14.3^$$$, ¤¤¤^

Values are expressed as mean and SD unless otherwise stated.

COPD = chronic obstructive pulmonary diseases; N/A = not applicable; kg = kilogram; m = meter; FEV_1_ = forced expiratory volume in 1 s; FVC = forced vital capacity; TLC = total lung capacity; RV = residual volume; DLco = carbon monoxide diffusing capacity; BAL = bronchoalveolar lavage; CRQ = chronic respiratory questionnaire; g = gram; L = liter. ¶ = only current smokers COPD.

^$^p ≤ 0.05, ^$$^p ≤ 0.01, ^$$$^p ≤ 0.001 for comparison with never smokers; ^¤^p ≤ 0.05, ^¤¤^p ≤ 0.01, ^¤¤¤^p ≤ 0.001 for comparison with smokers.

**Table 2:** Demographics, lung function and markers of systemic and local inflammation In COPD patients divided into current smokers and ex-smokers

**Variables**	**COPD (current smokers)**	**COPD (ex-smokers)**
N (male/female)	28 (15/13)	12 (5/7)
Age	58.9 ± 5.1	59.8 ± 5.9
Body mass index (kg/m^2^)	24.1 ± 3.8	27.7 ± 4.0^$^
Height (Centimetres)	172 ± 1.4	170 ± 2.5
Smoking (pack years)	41.6 ± 10.4	29.1 ± 8.9^$$$^
Cigarette per day (last 6 months)	16.4 ± 6.4	N/A
Time since smoking cessation years (range)	N/A	(2–16)
FEV_1_ (% predicted)	79.4 ± 10.8	78.1 ± 14.0
FEV_1_/FVC	0.61 ± 5.9	0.61 ± 7.8
DLco (% predicted)	66.3 ± 12.1	67.6 ± 15.3
TLC (% predicted)	107.6 ± 16.5	109.6 ± 10.4
RV (% predicted)	137.6 ± 36.9	138.8 ± 25.9
Chronic bronchitis (% of individuals)	25	17
Dyspnoea score (CRQ)	5.9 ± 0.91	6.1 ± 0.72
White blood cell counts (10^9^/L )	8.0 ± 1.9	7.0 ± 1.9
Serum -high sensitive-CRP (g/L)	3.2 ± 3.0	2.7 ± 1.9
Serum -orosomucoid (g/L)	0.86 ± 0.18	0.80 ± 0.12
Serum -haptoglobin (g/L)	1.5 ± 0.45	1.1 ± 0.36^$$^
Serum -immunoglobulin G (g/L)	9.4 ± 1.7	10.3 ± 2.7
BAL cell concentration (10^6^/L)	492.5 ± 255.3	118.6 ± 69.1^$$$^
BAL macrophages (10^6^/L)	470.9 ± 250.0	101.2 ± 64.0^$$$^
BAL lymphocytes (10^6^/L)	13.1 ± 9.0	14.4 ± 8.4
BAL neutrophils (10^6^/L)	4.7 ± 4.1	2.7 ± 2.1
BAL eosinophils (10^6^/L)	3.5 ± 1.9	0.23 ± 0. 13
BAL recovery (%)	43.7 ± 14.8	50.4 ± 12.2

Values are expressed as mean and SD unless otherwise stated.

COPD = chronic obstructive pulmonary diseases; N/A = not applicable; kg = kilogram; m = meter; FEV_1_ = forced expiratory volume in 1 s; FVC = forced vital capacity; TLC = total lung capacity; RV = residual volume; DLco = carbon monoxide diffusing capacity; BAL = bronchoalveolar lavage; CRQ = chronic respiratory questionnaire; g = gram; L = liter.

^$^p ≤ 0.05, ^$$^p ≤ 0.01, ^$$$^p ≤ 0.001.

### Lung density expressed as mean lung attenuation and %HDS

**Figure 1:**
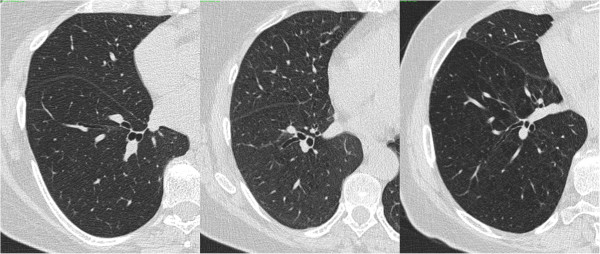
Axial inspiratory CT scans at the level of right inferior pulmonary vein, from three 54 year old females, representing typical patterns; Left panel: healthy never-smoker; Middle panel: smoker (note subtle uniform diffuse increased opacity which makes vessels more visible and mild interlobular septal thickening, especially in middle lobe; Right panel: COPD (note diffusely distributed centrilobular emphysema, more in central part of scans, with radiolucency and irregular vascular pattern).

**Figure 2:**
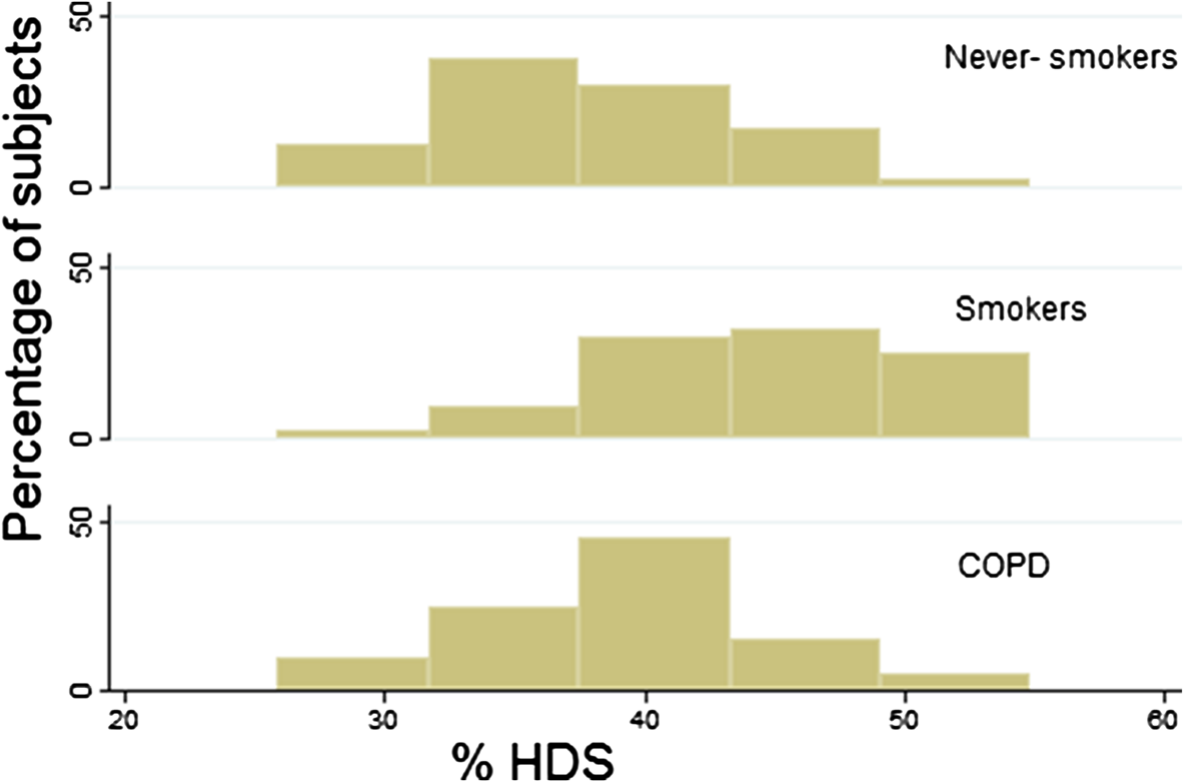
Histogram showing relationship between lung density measured as attenuation −750 HU to −900 HU (%HDS) for (never-smokers, smokers and COPD patients).

**Figure 3:**
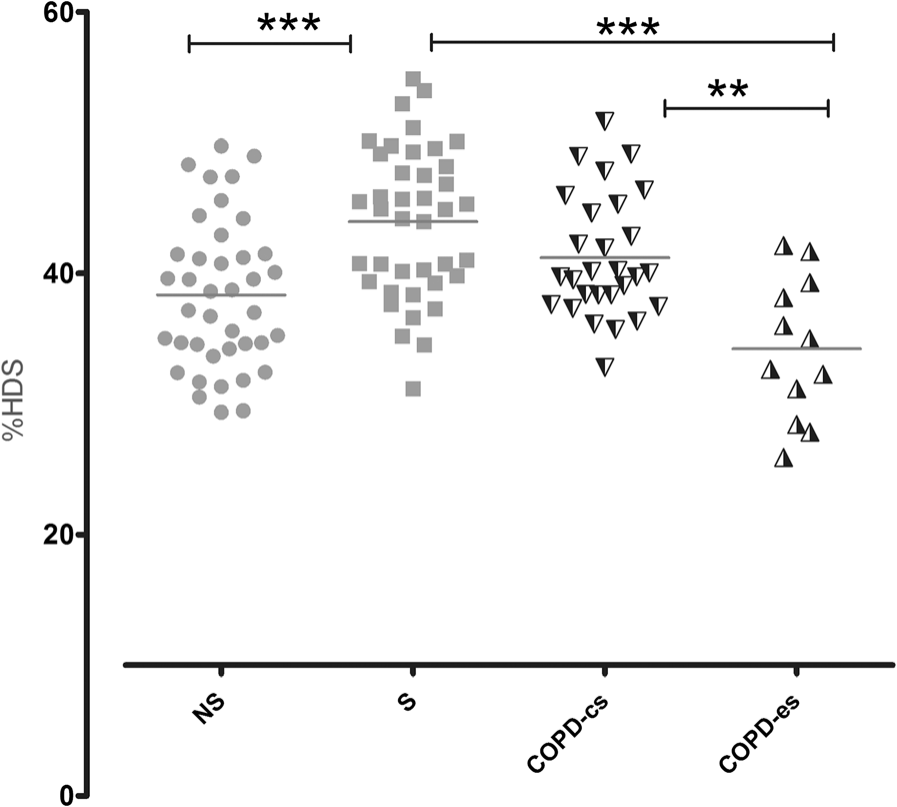
Lung density, measured as percentage of lung volume with attenuation −750 to −900 HU (%HDS) for never-smokers (NS), smokers (S), COPD current smokers (COPD-cs) and COPD ex-smokers (COPD-es).

Smokers had denser lungs than both never-smokers and COPD patients (Figure 1) resulting in a shift of lung attenuation values to a higher density (Figure 2). The mean lung attenuation was higher for smokers (−857 ± 28 HU; mean ± SD) than for never-smokers (−874 ± 20 HU) and patients with COPD (−876 ± 17 HU), (p < 0.001 for both). This was also reflected when lung density was quantified as %HDS; smokers (44.0 ± 5.8%), never-smokers (38.3 ± 5.8%) and COPD patients (39.1 ± 5.8%), (p < 0.001 for both), (Figure 3). There were no significant differences in lung density between the entire group of COPD patients and never-smokers.

When the COPD patients were divided into current smokers and ex-smokers, there was no difference in %HDS between COPD current smokers and smokers with normal lung function (p = 0.2). However, %HDS was significantly lower in COPD ex-smokers (33.1 ± 4.5%) compared to both COPD current smokers and smokers (p = 0.04 for both).

Areas with a density of −300 to −750 HU were calculated separately since this may represent pixels including non-parenchymal tissue such as bronchi and vessels. The percentage of lung in this spectrum was not significantly different between smokers (13.9% ± 6.1), never-smokers (12.5% ± 4.6) and COPD patients (11.1% ± 2.2).

### Difference in lung density between genders

**Figure 4:**
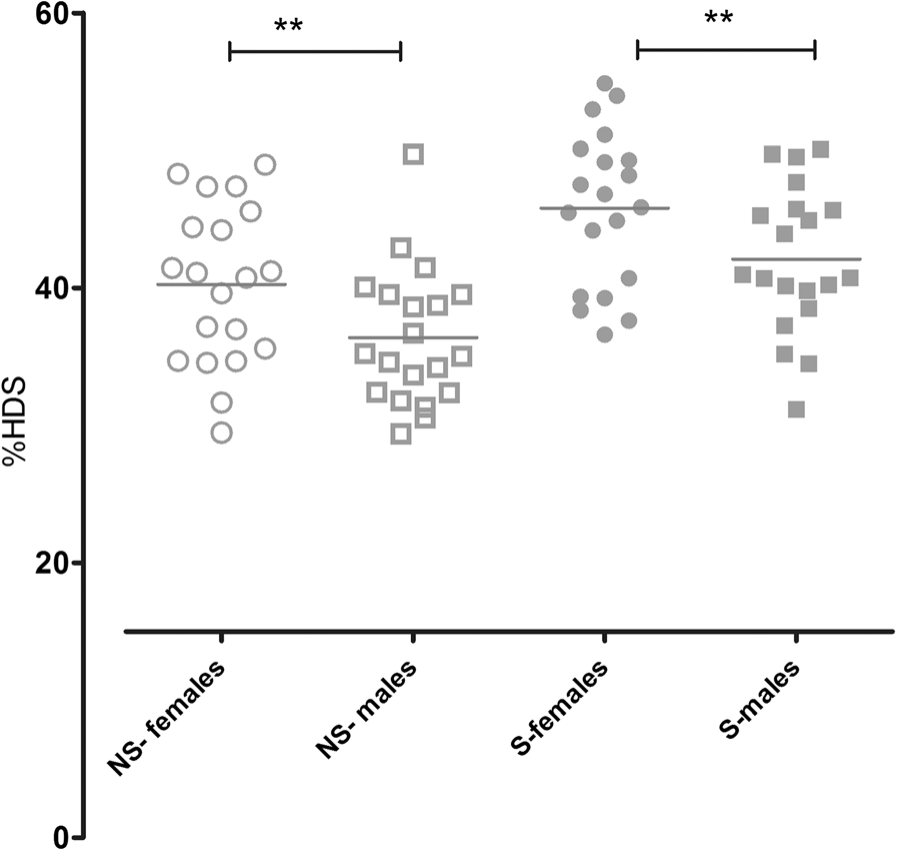
Lung density, measured as percentage of lung volume with attenuation −750 to −900 HU (%HDS) for never-smokers (NS) and smokers (S) separated into males and females.

Never smoking and smoking females had higher lung density measured as %HDS, (40 ± 1.3%) and (46 ± 1.1%) respectively compared to never-smoking and smoking males (36 ± 1.3%) and (42 ± 1.2%) and (p < 0.03 and p < 0.04), (Figure 4). There was no significant difference in lung density between males and females in the COPD group (data not shown). In a multiple regression model, after adjustment for height and total lung capacity these differences between genders were not able to demonstrate (data not shown).

### Correlations between lung density and measures of systemic inflammation

White blood cell count was significantly higher both in smokers and in COPD-patients compared to never-smokers as were the concentrations of orosomucoid and haptoglobin in serum. C-reactive protein was, however, significantly increased only in the COPD patients (Table 1). The concentration of Immunoglobulin G was lower in current smokers and COPD patients. Current smoking COPD patients had significantly higher concentration of haptoglobin than COPD ex-smokers (Table 2). There was a significant positive correlation between lung density (%HDS) and haptoglobin in serum in both smokers and COPD patients, while immunoglobulin G had negative correlation in smokers and COPD (Table 3). There was no significant correlation between lung density and other markers of systemic inflammation measured in serum.

**Table 3:** Pearson correlations coefficients between %HDS and differential cell count in BAL, serum proteins, lung function, smoking status and demographic data

**Variables**	**Never smokers**	**Smokers**	**COPD**
BAL cell concentration (10^6^/L)	-	0.38^$^	0.57^$$$^
BAL macrophages (10^6^/L)	-	0.36^$^	0.57^$$$^
BAL lymphocytes (10^6^/L)	-	0.41^$$^	-
BAL neutophils (10^6^/L)	-	0.47^$$^	0.40^$^
Serum-haptoglobin (g/L)	-	0.37^$^	0.32^$^
Serum-immunoglobulin G (g/L)	-	−0.50^$$^	−0.41^$^
TLC (% predicted)	−0,52^$$$^	-	−0.39^$^
RV (% predicted)	−0,33^$^	-	−043^$$^
FEV_1_ (% predicted)	-	-	-
DLco (% predicted)	-	-	-
Smoking history (pack years)	-	-	-
Cigarette per day (last 6 months)	-	-	0.41^$$^
Height	−0.40^$$^	−0.32^$^	-

COPD = chronic obstructive pulmonary diseases; BAL = bronchoalveolar lavage; L = liter; g = gram; FEV_1_ = forced expiratory volume in 1 s; TLC = total lung capacity; RV = residual volume; DLco = carbon monoxide diffusing capacity; BMI = body mass index.

Values are expressing correlations coefficients.

^$^p ≤ 0.05, ^$$^p ≤ 0.01, ^$$$^p ≤ 0.001.

- Not statistically significant.

### Correlations between lung density and measures of local inflammation

Total cell and macrophage concentrations in BAL were higher in current smokers than in never-smokers and COPD ex-smokers (Table 1). The BAL-recovery, measured as percentage of recovered fluid, was significantly higher in female smokers with normal lung function than in their male counterparts. There were no any other significant differences between males and females in any other BAL parameter (data not shown).

The increase in cell concentration in the COPD group was mainly determined by COPD current smokers (Table 2). The concentration of neutrophils did not differ between smokers with normal lung function and COPD patients, although both groups had higher concentration than never-smokers.

**Figure 5:**
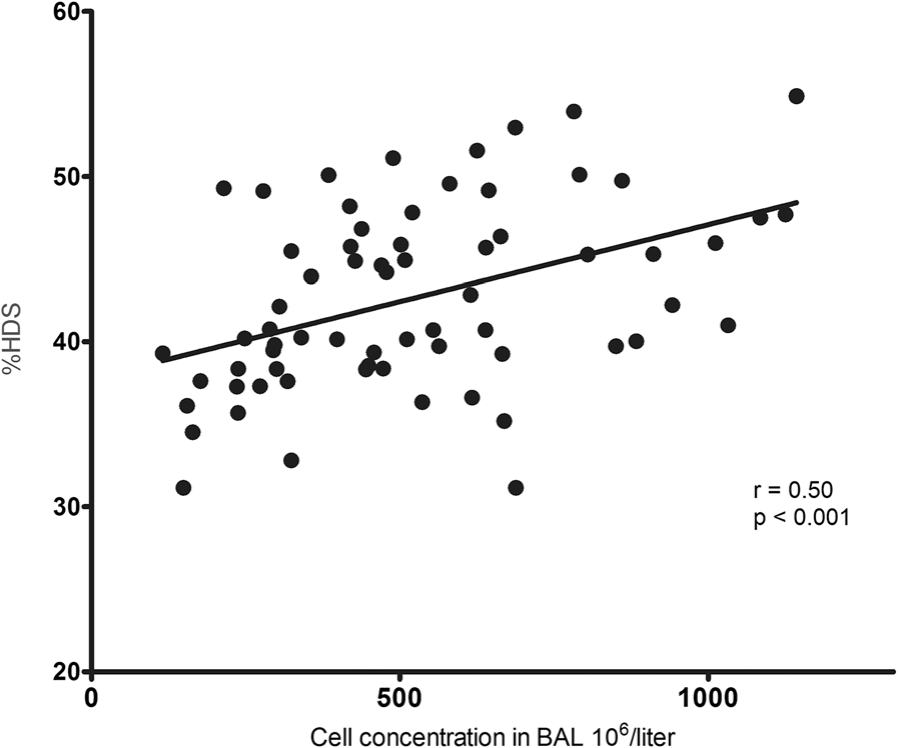
Correlation between percentage of lung volume with attenuation −750 to −900 HU (%HDS) and cell concentration in BAL for current smokers.

There was a positive correlation between lung density (%HDS) and total cell concentration in BAL both for smokers with normal lung function (r = 0.38, p = 0.03) and when smoking COPD patients were included (r = 0.50, p < 0.001) (Table 3 and Figure 5). There was no correlation between cell concentration in BAL and lung density in never-smokers.

### Correlations between lung density and lung function/smoking history, BMI, age and height

In the COPD patients, total lung capacity (TLC) and residual volume (RV) were both negatively correlated to lung density measured as %HDS (Table 3). In a multivariate model, TLC showed a significant negative association with lung density in never-smokers (R^2^ = 31, parameter estimate −0.95; 95% CI −0.70 to −0.11; p = 0.007) and in COPD patients (R^2^ = 64; parameter estimate −0.17; 95% CI −0.28 to −0.07; p = 0.001) but not in smokers with normal lung function. Current cigarette consumption, measured as numbers of cigarettes per day, was correlated to %HDS, (r = 0.41; p = 0.008), but there was no correlation to cumulative smoking history expressed as pack years. %HDS was negatively correlated to height in never-smokers and smokers but not in COPD patients. Age and BMI did not correlate to lung density.

### Correlation between lung volumes measured by CT and body plethysmography

**Figure 6:**
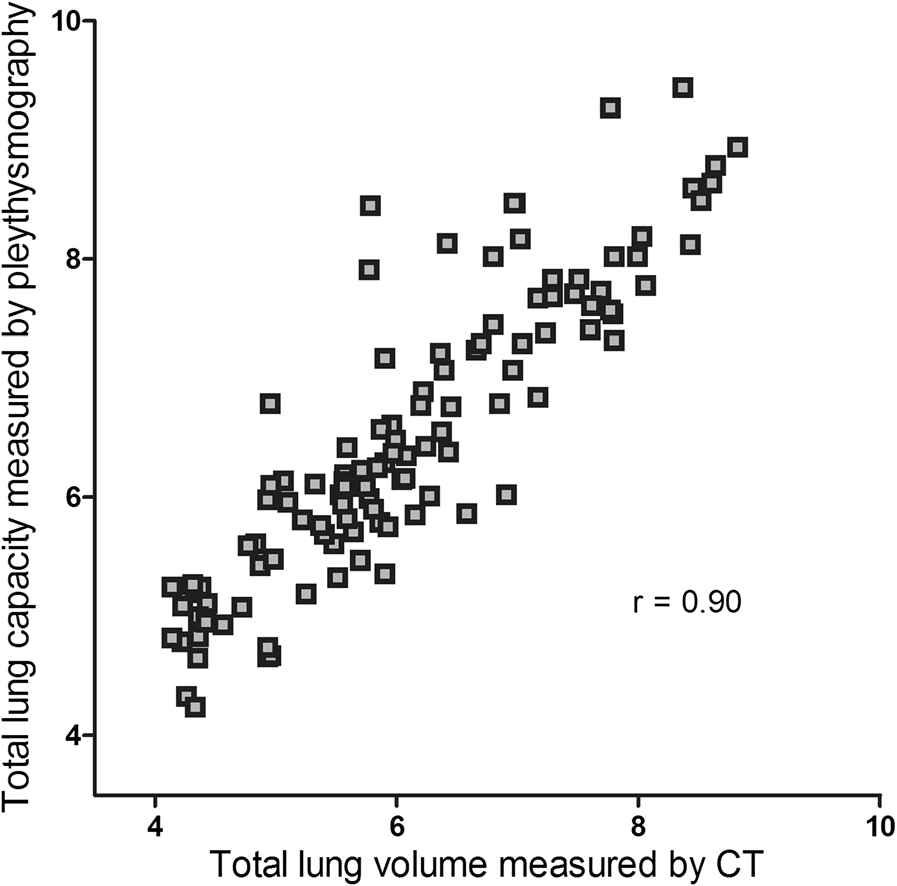
Relationship between total lung volume measured by CT and total lung capacity measured by body plethysmography.

Agreement between lung volumes measured by CT and body plethysmography is expressed graphically in Figure 6 for all subjects; the correlation coefficient was 0.9 (p < 0.001). There were no significant difference regarding level of inspiration between never-smokers, smokers and COPD-patients.

## Discussion

In this study we aimed to quantify the impact of chronic cigarette smoking on lung density on HRCT by studying smokers with normal lung function and COPD patients. We demonstrated that lung density was positively correlated to measures of local inflammation, i.e. the total cell concentration as well as concentrations of macrophages and neutrophils in BAL. In addition, females had denser lungs than males, regardless of smoking habits.

Earlier morphological studies on the lung of smokers have revealed that inflammation in small airways and lung parenchyma precedes architectural distortion and emphysema [8,16,22] and Remy-Jardin et al. [23] demonstrated that early subtle parenchymal inflammation in normal smokers correlated to development of emphysema. Our findings with increased lung attenuation in smokers are in line with previous reports [12,23-25], although few studies have been performed including healthy never-smokers. Lederer et al. [24] reported a positive correlation between lung density and cumulative and current smoking history. Shaker et al. [12] reported a significantly increased lung density in current smokers which decreased after smoking cessation [26]. During a 5-year follow-up, Soejima et al. [25] found that the density on inspiratory CT increased in smokers compared to never-smokers while it decreased after smoking cessation.

Cigarette smoking induces a dose-related inflammatory response in the lungs with accumulation of macrophages and neutrophils into the lower respiratory tract [27,28]. In agreement with these previous reports, we found that the numbers of macrophages and neutrophils were significantly raised in smokers with normal lung function and in current smoking COPD patients. The inflammatory process seems to be reversible, at least in terms of these parameters, since COPD ex-smokers exhibited cell number in BAL fluid equal to those of never-smokers.

We demonstrated a positive correlation between lung density and cell concentration in BAL fluid both in smokers with normal lung function and in current smoking COPD patients. Lung density does not solely depend on the presence of cells in the alveolar space and in the lung interstitium but may also depend on other factors such as oedema and peribronchiolar fibrosis. We consider, however, that cell concentration in BAL fluid could be regarded as an overall indicator of the inflammatory process in the lung. The fact that DLCO was reduced in smokers with normal lung function suggests an increased barrier for oxygen diffusion due to inflammation in the alveolar interstitium rather than reduced area for gas exchange due to emphysema, while the latter may be dominant in COPD patients.

An interesting finding is that lung density in our entire group of COPD patients was lower than the smokers’ and did not differ from never-smokers. A plausible explanation may be that areas with decreased lung density in COPD patients (i.e. emphysema) and areas with increased lung density (i.e. accumulation of inflammatory cells) may occur in the same patient but in different regions of the lung. This results in an average density in the COPD-patients’ whole lung, that does not differ from never-smokers. When the COPD patients were sub-grouped in ex-smokers and current smokers, the latter had higher density. This suggests that inflammation due to smoking (i.e. increased lung density) overshadows potential reduction in lung density due to parenchymal destruction. In COPD ex-smokers, the absence of smoke-induced cell infiltration in the lower airways results in a lower density, probably due to parenchymal destruction and emphysema. This hypothesis is also supported by our finding that total cell concentration in BAL fluid was significantly lower in COPD ex-smokers than in COPD current smokers.

It is known that smokers and COPD patients have a low grade of systemic inflammation indicated by elevated serum markers [2,29]. We found significantly higher levels of white blood cells, orosomucoid and haptoglobin but decreased level of immunoglobulin G in serum of smokers and COPD patients compared to never-smokers. In smokers and COPD patients, lung density was positively correlated with the concentration of haptoglobin in serum, but not to C reactive protein (CRP). Haptoglobin is an acute phase protein which is elevated in inflammatory and infectious conditions and may be a sensitive marker for low grade systemic inflammation. The levels of immunoglobulin G were significantly lower in smokers and in COPD current smokers compared with never-smokers, which reassures an immunosuppressive effect of cigarette smoking [30].

Previous reports have shown gender differences for lung function impairment and symptoms related to smoking [31-33]. Dransfield et al. [34] reported that the attenuation area lower than −950 HU on CT was greater in male than in female COPD patients, suggesting more severe emphysema in men. Other investigators have reported a more rapid progression of emphysema in females [12,32]. Gevenois et al. [35], did not find any gender differences when they measured mean lung attenuation in never-smokers. We found that women had significantly higher lung density than men regardless of smoking status, but the difference were no longer present when adjustment for height in multivariate models was made. Thus, the gender difference is probably anatomically related and suggests that women may have more lung tissue per volume compared to men. Smaller ribcage and shorter diaphragm in females are reported previously [36]. We could not observe any gender difference regarding BAL cell count or lung function parameter including DLCO. Lower percentage of BAL fluid recovery in male smokers may indicate different response to cigarette smoke between genders.

Our study has some limitations. The number of participants with COPD was low, which makes the subgroup analysis uncertain with regard to bronchitis and airways obstruction. The clinical utility of CT for characterization of airway obstruction in phenotypes with parenchymal destruction or mainly airway predominant disease has been promising in some studies. However there is a considerable overlap between these phenotypes especially in advanced stages of COPD [37,38]. The question rises if dividing our COPD patients in two main parenchymal and airway predominant phenotypes produce more correct correlation to inflammatory measurers in BAL in this group. We deliberately included only COPD patients of GOLD stage I and II to detect subtle parenchymal changes. Secondly, measurement of small airways i.e. with diameter less than 2 mm is difficult to perform accurately. The lower attenuation limit of −900 HU considered to be rational based on a report demonstrating pathological specimens from patients with emphysema comprise more voxels with attenuation lower than −900 HU [18]. Areas with a density of -300 to -750 HU were calculated separately since this may represent pixels including tissue such as small bronchi and vessels. Thus, we consider that areas with a density of -750 and -900 HU represent lung tissue with minimal interference from emphysema and denser structures and this area can represent a good surrogate for measuring the degree of local inflammation in the lung. The inflammatory process in smokers is mainly concentrated in conductive airways (with diameter less than 0, 5 mm), lung parenchyma and alveolar space [8,39] and cells recruited by BAL represent these areas. A lung attenuation spectrum on CT which may represent these corresponding areas is thus motivated. The fact that lung density in the spectrum between −300 HU and −750 HU did not differ between the three studied groups may indicate that the inflammatory changes with impact on lung density occur mainly in the conductive airways and in the lung parenchyma.

Lung density is influenced by a number of factors including the level of inspiration. To validate this important factor we compared the total lung volume measured by CT and the total lung capacity measured by body plethysmography. We found a good agreement in all three groups indicating that the level of inspiration had a negligible effect on our results. Lung density was negatively correlated to TLC but not age in never-smokers and this finding is in agreement with a study from Gevenois et al. [35].

The strength of our study is that we included a group of healthy never-smokers of both genders. Further, all measurements, including CT scans and BAL, were performed locally with same equipment and followed standardized procedures. We believe that our results can be valuable for selection of patients in research and clinical trials dealing with CT and smoking associated pulmonary diseases. By following attenuation trend inter-individually it may be possible to determine when the preceding inflammation “switches” to emphysema. The findings may help to interpret high resolution CT in the context of smoking and gender and highlight the heterogeneity of structural changes in COPD.

In conclusion, smokers have denser lungs than never-smokers and females have denser lungs than males. The density in smokers is associated to measures of systemic inflammation and cell concentration in BAL. The results support our hypothesis that smoking causes an inflammation in the lungs which can be quantified non-invasively by means of CT density.

## Competing interest

The authors declare that they have no competing interests.

## Authors’ contributions

Study design, RK, CMS, GT, SN, ÅW; Data collection, RK, CMS, MM, HF, SN; Data analysis and writing, RK, CMS, MM, GT, SN; Data interpretation, RK, CMS, GT, ÅW, SN; Critical review, RK, CMS, SN, MM, GT, ÅW, HF. All authors read and approved the final manuscript.
